# Fast, Comprehensive, and User Customizable Macromolecule Interface Analysis with FACE2FACE

**DOI:** 10.1021/acs.jcim.5c00771

**Published:** 2025-07-30

**Authors:** Patrizio Di Micco, Mario Incarnato, Gianmarco Pascarella, Allegra Via, Veronica Morea

**Affiliations:** † Institute of Molecular Biology and Pathology (IBPM), National Research Council of Italy (CNR), Rome 00185, Italy; ‡ Institute of Biomembranes, Bioenergetics and Molecular Biotechnologies (IBIOM), National Research Council of Italy (CNR), Bari 70126, Italy; § Department of Biochemical Sciences “A. Rossi Fanelli”, “Sapienza” University of Rome, Rome 00185, Italy

## Abstract

Structural analysis of interfaces in macromolecular complexes is crucial to unveiling the mechanisms underlying molecular recognition. While several valuable computational tools exist for interface analysis, many web-based tools have limitations in input types, analysis comprehensiveness, or output customization, and there remains a need for an immediately accessible solution requiring no software installation, especially for users with limited computational skills. We have developed FACE2FACE, a user-friendly, fast, and comprehensive tool available as a web server for macromolecule interface analysis. FACE2FACE analyzes interfaces between proteins, nucleic acids, and other biological macromolecules or small molecules, providing extensive information that can be instantly visualized on the server interface and easily downloaded. The downloaded materials comprise files in formats that can be easily parsed and imported in spreadsheet applications as customizable contact maps and scripts to quickly visualize interface features in widely used applications such as PyMol and ChimeraX. Examples of FACE2FACE contributions to research projects are described.

## Graphical abstract

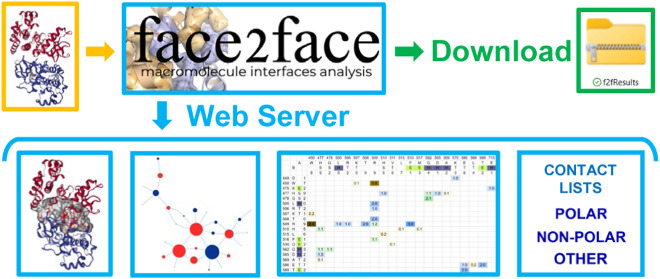

## 1 Introduction

Molecular recognition plays a crucial role in essential biological functions. To uncover the mechanisms driving this phenomenon, it is necessary to analyze the interfaces involving biological macromolecules. Understanding the properties of these interfaces increases our knowledge about protein sequence-structure–function relationships, factors affecting tertiary and quaternary structure stability, mechanisms of allosteric regulation and function, and molecular evolution and helps distinguish between transient and permanent complexes. A detailed understanding of the structural and functional roles of specific interface residues serves as the rational foundation to successfully design protein mutants or short peptides with desired functions, stability, or other properties. In structure biology, interface analysis is used to investigate quaternary interactions in multimeric structures solved by X-ray crystallography, electron microscopy, or nuclear magnetic resonance.

The significance of interface analysis in molecular biology has spurred the development of numerous valuable tools over the years. Several computational packages and toolkits have been developed for the analysis of molecular interfaces, including standalone software solutions that provide comprehensive analytical capabilities.^1–4^ Some tools are also freely available as web servers (e.g., refs 5–11 ). While these resources provide valuable functionalities, there remains a need for a comprehensive, immediately accessible web-based solution that supports interface analysis without requiring software installation, dependency management, or local computational infrastructure. Conversely, many existing tools demand local installation, system-specific configuration, or command-line proficiency, which can represent barriers for researchers with limited programming experience or restricted computing environments or in educational settings. Furthermore, current web-based tools often have limitations in the type of accepted input, performed analysis, or provided output. Typically, only one type of biological macromolecule, usually proteins, is accepted as input. In terms of analyses, interfaces are generally identified based on either interatomic distances or solvent-accessible surface area (SASA), but not both; interactions are reported at the residue level, but not at the atomic level, and their chemical nature is either unspecified or specified only for polar interactions; contact maps are not always included in the output; when they are, their format is noneditable, which can limit further analysis and data customization by the user. Moreover, not all existing web servers support interface visualization through integrated three-dimensional (3D) structure viewers or provide scripts to easily load coordinates with mapped interface features into structure visualization and analysis programs. In summary, to obtain all the information required for a comprehensive interface description in formats that allow further analyses to be rapidly performed, it is currently necessary to interrogate different web servers and use several additional programs to edit and customize their output.

These considerations led us to develop FACE2FACE, a user-friendly, fast, and comprehensive web-based tool designed to provide extensive information about interface features in formats that can be easily visualized and parsed by programs, such as text editors, spreadsheet applications, and structure analysis software.

## 2 Usage

### 2.1 Input Files

FACE2FACE accepts as input the atomic coordinates of proteins, nucleic acids, or small molecules. Coordinates can be supplied either entering a Protein Data Bank (PDB) identifier^12^ or uploading a file from local storage in standard PDB or mmCIF format.

Once a PDB identifier or coordinate file is selected, the server displays the 3D structure along with a list of all chains for user selection. Macromolecules are represented as ribbons, and each chain is assigned a distinct color to help identify those involved in the interface of interest. Small molecules are shown as balls-and-sticks, with colors indicating atom types: blue for nitrogen (N), red for oxygen (O), and gray for carbon (C). For proteins and nucleic acids, the “chain 1” and “chain 2” fields list all the chains present in the selected coordinate file for user selection; if the same chain is selected in both, new fields (i.e., “region 1” and “region 2”) appear, where users can input the residue numbers defining selected intrachain regions. This option is useful to investigate interfaces between domains, secondary structures, or other regions of interest that occur within the same chain.

When “biological unit” files are available on the PDB website, FACE2FACE lists all the chains present in each assembly and allows the user to select the assembly to investigate. If the input file contains an ensemble of NMR-determined structures, then the program automatically selects the first model in the file.

### 2.2 Output Files

The program output is rapidly visualized on the web interface and can be downloaded as a compressed (zipped) folder. The folder contains the following file sets:

i. **Contact lists** (.txt format). For macromolecules, three separate files list polar/polar (including hydrogen and ionic bonds), nonpolar/nonpolar, and polar/nonpolar contacts between the selected interface regions (Figure 1). Each file specifies the type and number of residues, the type of atoms involved in each contact, and the distance between them. For small-molecule ligands, a single file is provided without specifying the contact nature. Both types of files are available for download and can be viewed on the server.
ii. **Contact map files** (.csv format, ready to be imported as a matrix in spreadsheet software, and .xlsx format, ready to be opened in Microsoft Excel). A “light” matrix file includes only residues involved in interface contacts, whereas a “full” matrix comprises all residues within the regions selected as input. Chain name, residue number, residue type, and interface surface area are displayed for proteins and nucleic acids, and secondary structure (H: α-helix; S: β-strand) for proteins. Each cell corresponding to two interacting residues is colored based on the interactions that they establish (polar only, blue; only hydrophobic, yellow; both polar and hydrophobic, green) and the number of interactions (the higher the number, the darker the color). Within these cells, the number of polar and nonpolar contacts are reported as integer numbers followed or preceded by a dot, respectively (see Figure 1). SASA burial values of each residue are also reported. These can help evaluate the relevance and quality of contacts, as extensively buried interfaces usually correspond to strong and favorable interactions, while minimally buried interfaces may indicate surface-exposed or incidental proximities. If one of the interface partners is a small-molecule ligand, the type of each atom of the small molecule and of the macromolecule residue interacting with it are shown; the cells corresponding to interacting atoms display the distances between the interacting atoms in Å; and the cell background is colored gray, with the intensity increasing as the distance decreases. Users can easily edit spreadsheets contents to add data, remove unnecessary information, and create customized pictures. Light contact maps are available for download and visible on the server, whereas full contact maps can only be downloaded.
iii. **Ready-to-run scripts** for quick interface visualization are available for PyMol (The PyMOL Molecular Graphics System, Version 3.0 Schrödinger, LLC) and ChimeraX,^13^ two of the most used programs for 3D structure visualization and analysis. In both programs, chains are displayed as ribbons, with each chain in a different color (Figure 1). Additionally, residues in interface regions are shown as sticks, highlighted by their solvent-accessible surface and colored by atom type (nitrogen in blue, oxygen in red, and carbon in the same color as the chain they belong to). Both PyMol and ChimeraX offer numerous options to customize the default view and perform further analysis. These scripts are available for download; additionally, the server immediately updates the view of the 3D structure given as input to show interface-related information similar to what is described above: only the chains, or chain, comprising interface residues are shown, with different chains in different colors: their solvent-accessible surface area is displayed; and interface residues are highlighted by ball-and-stick representation and colored according to the chain, or intrachain region, they belong to.

**Figure 1:**
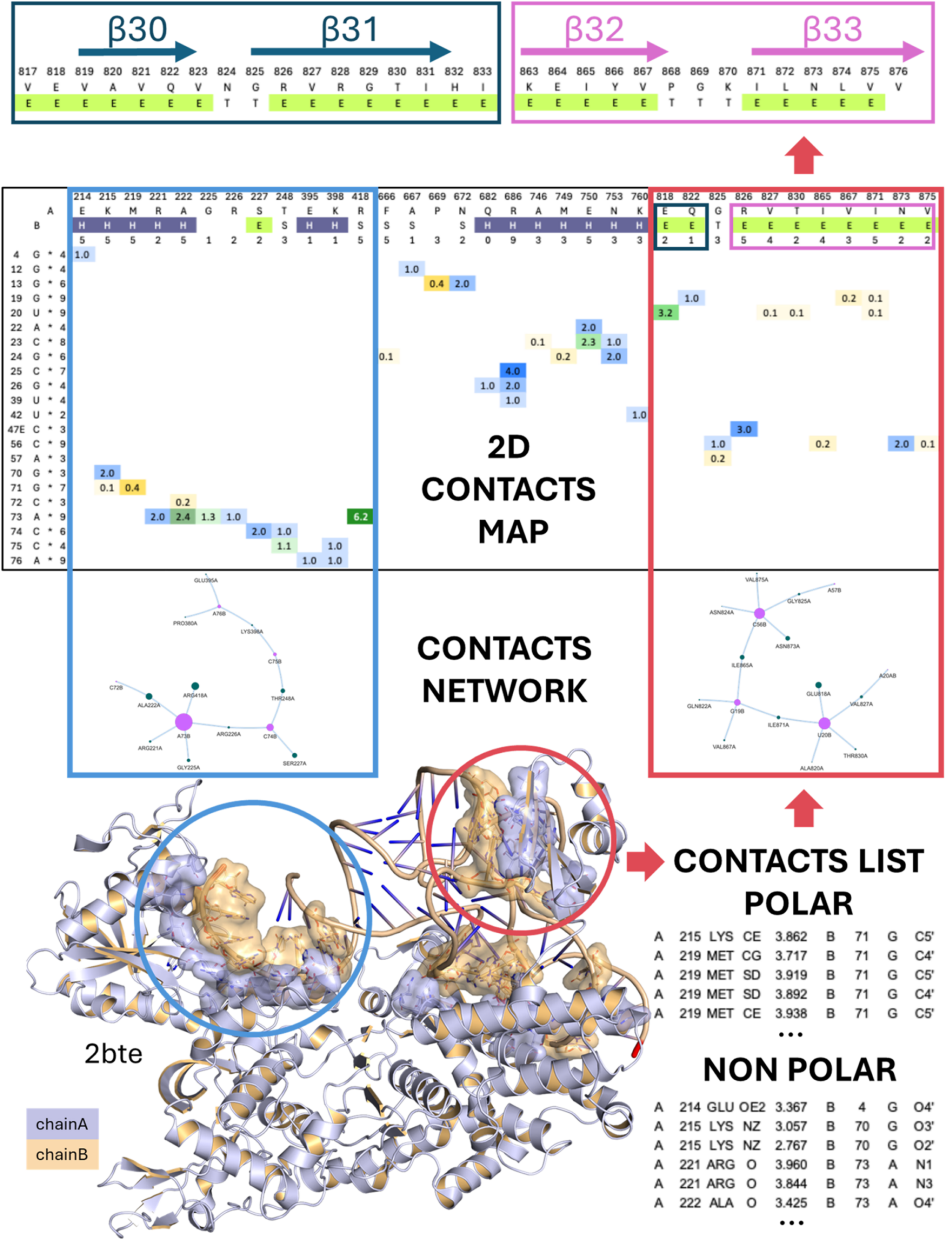
FACE2FACE analysis of the interface between *Thermus thermophilus* leucyl-tRNA synthetase (LeuRS) and tRNA^Leu^. The bottom-left part of the image shows the 3D structures involved in interface regions in the LeuRS-tRNA^Leu^ complex from *Thermus thermophilus* (PDB ID: 2BTE). The chains of the two interacting macromolecules are shown as ribbons (chain A: LeuRS, lilac; chain B: tRNA^Leu^, light orange). Interface residues are shown as sticks colored by atom type: N, blue; O, red; C and solvent-accessible surfaces are lilac or light orange, depending on whether they belong to chain A or B. This is the default representation generated by the ready-to-run downloadable script for PyMol, and a similar representation is available on the server. Blue and red circles highlight interface regions for which contact networks and maps are shown in the panels above, within frameworks with the same colors. The bottom-right part of the image shows examples of contacts files comprising list of interactions between polar atoms only and nonpolar atoms only, respectively. These files, along with a file for contacts between polar and nonpolar atoms, are available on the server and for download. The “contacts network” part of the image represents interface residues as nodes. Node size is proportional to the number of interface contacts, and color indicates the chain they belong to. This feature is available only on the web server, where users can move nodes interactively for a clearer view. The “2D contacts map” part of the image reports the name of each interacting chain, and the name, number, secondary structure, and solvent-accessible surface area (SASA) buried at the interface of each residue. Colored cells indicate residue contacts involving specific atom types (i.e., all polar: blue; all hydrophobic: yellow; both polar and hydrophobic: green), the color shade being darker for higher number of contacts. The number of polar and nonpolar contacts between residues are shown as integers within these cells, and are followed and preceded by a dot, respectively. These 2D maps are available on the server and for download. The top part of the image shows the sequences of two LeuRS regions comprising β strands 30–31 and 32–33 within the dark blue and magenta framework, respectively. Based on the high number of contacts between these regions and tRNA^Leu^, isolated peptides with the same sequences, named β30_31 and β32_33, respectively, were selected as potential tRNA^Leu^ binders.

In addition to the output described above, the web interface allows an **interactive network of contact residues** to be visualized, which is not present in the downloadable folder. In this network, each residue is represented by a node, whose color indicates the chain to which it belongs and whose size reflects the number of interactions it has with other residues. Additionally, users can interactively move nodes to get a clearer view of the interface interactions. This interaction network can help users rapidly identify interaction hot spots.

### 2.3 Users Support

FACE2FACE offers user support through two main channels. Every page includes links to both the “Help” and “Contacts” sections. The “Help” page provides a detailed, step-by-step guide on how to proficiently use the platform. The “Contacts” page provides a form that allows users to submit questions, comments, feedback, and suggestions, which will help us to enhance the server’s effectiveness and usability.

## 3 Methods

Once two chains or chain regions are selected, FACE2FACE calculates both interatomic distances and solvent-accessible surface area (SASA) buried at the interface of each residue within the selected regions. Additionally, the secondary structure of each residue is obtained using the DSSP program.^14^

Two atoms are considered to be in contact if their distance is less than or equal to a user-defined threshold (default value: 4.0 Å). Contacts are classified as “polar” if they involve only N or O atoms, “nonpolar” if they involve only C or S atoms, and “other” if they involve one N or O atom and one C or S atom. This simple definition, based solely on interatomic distance and atom type, without considering angles,^15^ compensates for potential inaccuracies in experimental structures solved at low resolution or in computational models. Since in most structures determined by X-ray crystallography hydrogen atoms are not present, they would have to be computationally added to allow hydrogen bond angles to be measured. This can be done only in some cases on the basis of stereochemical rules (i.e., H atoms linked to sp^2^ hybridized heteroatoms, such as ND2 of Asn or NE2 of Gln; NE, NH1, and NH2 of Arg; and main-chain N atoms), whereas in the case of sp^3^ hybridized heteroatoms (e.g., NE of Lys or OH of Tyr), hydrogen atom positions are assigned arbitrarily. Additionally, when structure resolution is not high enough to distinguish between hydrogen-bound ND2 and hydrogen-free OD2 atoms in the side chain of Asn residues or between hydrogen-bound NE2 and hydrogen-free OE2 atoms in the side chain of Gln residues, in the absence of obvious hydrogen bond partners, the relative position of side-chain N and O atoms in Asn and Gln residues is chosen arbitrarily as well. For these reasons, a lenient criterion for polar interactions was chosen, allowing users to perform more detailed investigations, if needed.

In case the interface comprises a small-molecule ligand, its atoms are not classified as polar or nonpolar and all interatomic contacts are listed together. Since FACE2FACE focuses on biological macromolecules, detailed polarity descriptions of the atoms belonging to small molecules interacting with a biological macromolecule are beyond the scope of this tool and are left to the user’s knowledge of the system under investigation and methods specialized in small-molecule analyses.

Interface buried SASA is calculated from the difference between the SASA value of each residue in the free state and in the complex, both of which are obtained using the program Naccess.^16^ Interface buried SASA values are expressed as integers from 0 to 9, representing ranges from ≤ 9 Å^2^ to ≥90 Å^2^, as previously reported.^17^

It is important to note that contacts identified solely by distance criteria do not necessarily represent favorable interactions. Residues may be in close proximity due to geometric constraints imposed by neighboring stabilizing interactions and may even experience repulsive forces while being held in place by the overall structure, giving rise to the so-called “frustrated interactions”.^18^ The integration of distance-based contacts with SASA burial results provides a broader picture given that extensive SASA burial typically indicates favorable interactions, whereas distance-based contacts lacking substantial SASA burial may reflect incidental proximity. Therefore, contact data should be interpreted in the context of chemical complementarity, structural environment, and, when available, supporting experimental evidence.

After these values have been calculated, contact-based interactive networks, two-dimensional (2D) contact maps, and scripts for PyMol and ChimeraX are generated.

FACE2FACE is written in Python, utilizing the SciPy library. The NGL library is used to display structures on the web server (https://github.com/nglviewer/ngl).^19,20^

This website is free and open to all users, and there is no login requirement.

## 4 Applications

Since its development, FACE2FACE has been leveraged in several research projects^21–25^ to facilitate interface analysis and peptide design.

The ability of FACE2FACE to quickly provide comprehensive data on macromolecular interfaces has been particularly valuable in analyzing the multiple interfaces present in the 3D structures of *Schistosoma mansoni* peroxiredoxin I determined by X-ray crystallography. This is a moonlighting protein that exists in two forms, with different quaternary assemblies and functions: the low-molecular-weight 10-mer, which exerts peroxiredoxin activity, and the high-molecular-weight 20-mer, which possesses chaperone activity. Comparison of the contact maps provided by FACE2FACE for all pairs of interfacing subunits in the structure of the 10-mer and of the 20-mer has helped to reveal the mechanism by which chemical stressors induce the tertiary and quaternary variations that determine the transition between the two peroxiredoxin I forms.^24^

The interface data provided by FACE2FACE, and in particular, contact networks and 2D contact maps, can be leveraged to design peptides able to mimic the ability of one macromolecule to bind another. In this framework, we took advantage of FACE2FACE to successfully design peptides that can rescue the pathological effect caused by point mutations in mitochondrial (mt)-tRNAs in cell models. These point mutations are responsible for devastating diseases for which no therapy is currently available.^26^ Overexpression of mt proteins encoded by the nucleus, such as elongation factors and aminoacyl-tRNA synthetases (aaRSs), has been known to rescue pathological phenotypes associated with these mutations in cell models,^27–30^ presumably by acting through a chaperone-like mechanism.^31^ Among these proteins, leucyl-tRNA synthetase (LeuRS) is particularly attractive, since it is capable of rescuing the pathological phenotypes of cells bearing point mutations not only in the cognate mt-tRNA^Leu(UUR)^, but also in noncognate mt-tRNAs, such as mt-tRNA^Ile^, mt-tRNA^Val^, and mt-tRNA^Lys^.^31–33^ However, human mt-LeuRS is a very large enzyme, comprising over 900 amino acids, the use of which as a therapeutic agent would present many challenges. For this reason, we sought to develop short peptides (i.e., with length ≤ 16 a.a.), mapping on the LeuRS surface and endowed with the same rescuing ability as the whole enzyme. Since no structure of the complex between eukaryotic mt-LeuRS and mt-tRNA^Leu(UUR)^ is available from the PDB, we searched the NCBI database of proteins of known structure using the Blast program.^34^ *Thermus thermophilus* and *Escherichia coli* LeuRSs were identified as the closest human mt-LeuRS homologues, the structures of which had been determined in complex with tRNA^Leu^ (*E*-values: 8e^–175^ and 8e^–168^, respectively; %ID: 36% with both proteins). Contact networks and 2D contact maps of the LeuRS-tRNA^Leu^ interfaces present in these structures generated by FACE2FACE allowed us to quickly identify the LeuRS regions where the highest density of contacts with tRNA^Leu^ occurred, which corresponded to four β strands (i.e., β30, β31, β32, β33) within the C-terminal domain (Figure 1).^33^ Based on this information and on the conservation between the bacterial and human enzyme in these regions,^33^ we designed two peptides, named β30_31 and β32_33, each encompassing two of the four β strands. These peptides were subsequently demonstrated to possess the same rescuing ability as the entire mt-LeuRS in both yeast^35^ and human cells.^22,33^ They were then used to develop peptide-mimetic agents (WO 2023/126751 A1), which are currently undergoing in vivo studies to assess their therapeutic potential.

## 5 Conclusions

FACE2FACE is a novel, user-friendly, and fast tool that provides comprehensive information about interactions occurring in the 3D structures of complexes between proteins or nucleic acids, and other biological macromolecules or small molecules. This information includes interatomic distances and contact type (i.e., polar vs nonpolar), as well as the extent of solvent-accessible surface area of each residue that is buried in the interface. While other existing tools provide some of this information, FACE2FACE is the only server that offers all of them, both integrated into the server and in a downloadable format. Most importantly, unlike other tools, FACE2FACE provides interface information in formats that are easy to handle and ready for import in popular programs for data analysis and structure visualization such as spreadsheet applications for contact maps and PyMol/Chimera for 3D structures. This allows interface information to be further edited and customized, according to user’s requirements. Finally, by providing a single, web-based platform accessible through any browser and featuring a user-friendly interface, FACE2FACE removes potential usability barriers, since it requires no installation, prior computational expertise, or specific hardware resources and offers itself as a valuable resource for both research and educational communities.

Taken together, FACE2FACE features make it a particularly valuable web server for interface comparison, peptide design, and identification of hot-spot contact regions. Indeed, the program has already been successfully employed to investigate the assembly and disassembly mechanisms in proteins with highly multimeric quaternary structures and to pinpoint key interface regions for the design of peptides that mimic the activity of one of the macromolecules involved in the complex. Additionally, FACE2FACE results can aid the assessment of models of multimeric proteins or biological complexes provided by docking methods and the design of mutations that endow proteins with desired binding properties.

Future developments of FACE2FACE will focus on accommodating potential future variations in data formats and delivering increasingly efficient and accessible tools for the analysis of macromolecular interfaces.

## Data Availability

The FACE2FACE web server is freely available at https://face2face.ibpm.cnr.it. The data used in this study are freely available from the Protein Data Bank (PDB ID: 2BTE).
